# Cooperative investment in public goods is kin directed in communal nests of social birds

**DOI:** 10.1111/ele.12320

**Published:** 2014-07-06

**Authors:** René E van Dijk, Jennifer C Kaden, Araceli Argüelles-Ticó, Deborah A Dawson, Terry Burke, Ben J Hatchwell

**Affiliations:** Department of Animal and Plant Sciences, University of SheffieldWestern Bank, Sheffield, S10 2TN, UK

**Keywords:** cooperation, kin selection, public goods, social network theory, tragedy of the commons

## Abstract

The tragedy of the commons predicts social collapse when public goods are jointly exploited by individuals attempting to maximize their fitness at the expense of other social group members. However, animal societies have evolved many times despite this vulnerability to exploitation by selfish individuals. Kin selection offers a solution to this social dilemma, but in large social groups mean relatedness is often low. Sociable weavers (*Philetairus socius*) live in large colonies that share the benefits of a massive communal nest, which requires individual investment for construction and maintenance. Here, we show that despite low mean kinship within colonies, relatives are spatially and socially clustered and that nest-building males have higher local relatedness to other colony members than do non-building males. Alternative hypotheses received little support, so we conclude that the benefits of the public good are shared with kin and that cooperative investment is, despite the large size and low relatedness of these communities, kin directed.

## Introduction

Understanding how individuals resolve conflicts over contributions to or exploitation of common resources remains a major challenge in ecological research. The underlying public goods dilemma of payoffs from social benefits being generally highest when individuals cooperate, while selfish individuals do better than cooperators within groups, presents a temptation to defect and hence an evolutionary paradox (Rankin *et al.* 2007; Frank 2010; Drescher *et al.* 2014). This is exemplified in game theory by the Prisoner's Dilemma and more generally by the tragedy of the commons (Hardin 1968), a phenomenon that can be found in some form in virtually all biological systems, from unicellular organisms to our sophisticated societies (Houston *et al.* 2005; MacLean & Gudelj 2006; Rankin *et al.* 2007; Gutierrez *et al.* 2011; Drescher *et al.* 2014). Nevertheless, sociality has evolved many times, so mechanisms to resolve the conflict between selfish interests and social cooperation must be widespread.

An individual's decision of whether to cooperate or to defect depends critically on their social environment (Székely *et al*. 2010; van Dijk *et al.* 2012; Royle *et al.* 2012). For example, when social interactions occur among relatives, kin selection may tip the balance in favour of cooperation; indeed, kin-selected fitness gains have been invoked as a key driver in transitions to sociality in many taxa (West *et al.* 2002; Bourke 2011; Lukas & Clutton-Brock 2012; Fisher *et al.* 2013). Such interactions usually occur within relatively small, discrete groups composed largely of nuclear or extended family members, and many studies have revealed kin-biased cooperation in this context (e.g. Griffin & West 2003; Dickinson & Hatchwell 2004; Cornwallis *et al.* 2009). However, explaining cooperative investment in public goods is more challenging in large social groups with low average relatedness. One potential solution to this puzzle is that groups exhibit fine-scale kin structure and that cooperative behaviour is directed towards relatives through active or passive discrimination between kin and non-kin; indeed there is growing evidence for kin-directed behaviour in such situations (Hatchwell 2010). A study on Galápagos sea lions (*Zalophus wollebaeki*), for example, found that social ties between adjacent individuals in a network were positively related to genetic similarity (Wolf & Trillmich 2008). However, another of the few studies that have investigated genetic sub-structuring of large social groups found no evidence that genetic relatedness predicted social interactions in guppies (*Poecilia reticulata*; Croft *et al.* 2012). In general, there is a paucity of studies describing the association between group size and composition and the benefit that group membership confers (Shen *et al.* 2014).

**Figure 1:**
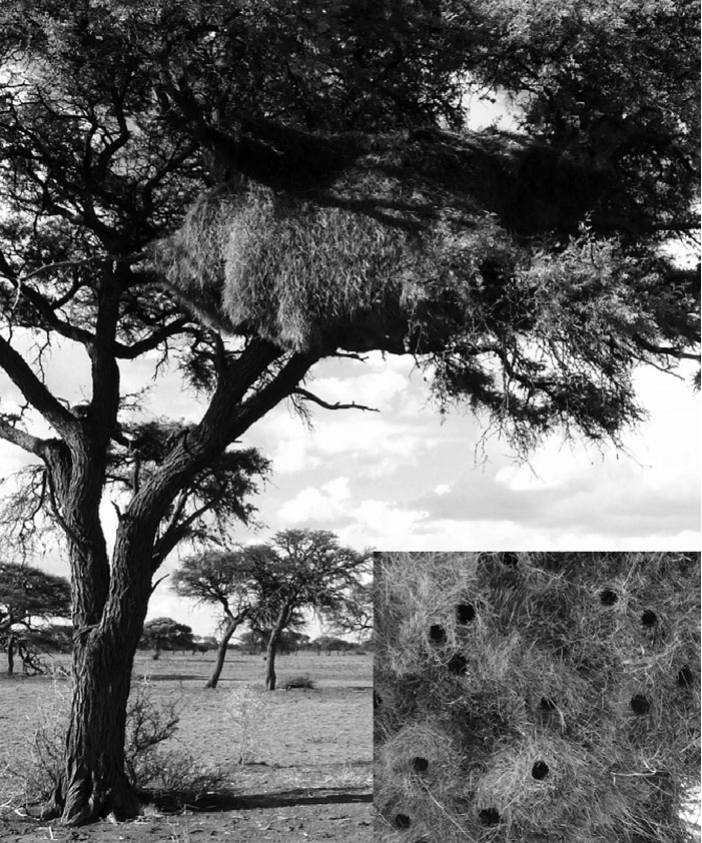
Example of a communal nest of sociable weavers. Inset: The downward-pointing nest chambers as seen from underneath the nest. Photographs by REvD.

In this study, we test the hypothesis that cooperative investment in the massive, communal nests of sociable weavers (*Philetairus socius*) is favoured when the benefits are shared with kin. The communal nest is larger than that of any other bird (Fig. 1) and comprises a communal thatch within which individual nest chambers are embedded, and it provides a social benefit by buffering variation in ambient temperature (van Dijk *et al.* 2013). The extent of this thermoregulatory benefit depends on the depth of the thatch, so that nest chambers which are more deeply embedded within a thatch, usually towards the centre of the communal nest, benefit from a larger thermoregulatory buffer, while the buffering effect for nest chambers near the edge is lower (van Dijk *et al.* 2013). The communal nest also offers protection as a predator refuge and provides a communal structure to support individual nest chambers (Collias & Collias 1977). Importantly, the thatch does not emerge from construction of nest chambers, but is a distinct structure requiring construction and maintenance. Thus, the communal thatch is an example of a public good requiring individual investment and conferring shared benefits. The potential tragedy of the commons in sociable weavers is the collapse of the communal nest mass, or parts of it, as a result of too little cooperative investment in thatch-building. This may affect all members of the colony, or smaller groups of individuals when parts of the nest collapse due to poor maintenance.

Males are the more philopatric sex in sociable weavers (Covas *et al.* 2006). Such population viscosity may lead to the emergence of kin neighbourhoods and is often the precursor to kin-directed cooperative behaviour in kin-structured social groups (Dickinson & Hatchwell 2004; Hatchwell 2009). However, the communal nests of sociable weaver may house hundreds of birds, so colony-level kin structure is low compared to the nuclear or extended family groups in which kin selection often operates (Bourke 2011; Lukas & Clutton-Brock 2012). The only previous study to investigate the effect of kinship on investment in communal nests found no evidence that relatedness between colony members of the eusocial wasp (*Ropalidia marginata*) predicted cooperative nest-building (Arathi & Gadagkar 1998).

Here, we use genetic analysis combined with behavioural observations to determine how a potential tragedy of the commons is averted. First, we investigate whether sociable weaver colonies have spatial or social substructure that is predicted by relatedness, thus forming fine-scale kin neighbourhoods within communal nests and creating an opportunity for ‘cryptic’ kin selection to operate (Hatchwell 2010). The extent to which such kin neighbourhoods may promote cooperation has hitherto been investigated primarily at landscape scale and in relation to cooperative breeding (Hatchwell 2009). We then test the hypothesis that the existence of fine-scale kin neighbourhoods within communal nests promotes cooperative investment in public goods.

## Materials and Methods

### Field methods

The sociable weaver is a colonial, cooperatively breeding passerine endemic to the semi-arid Acacia savannahs of southern Africa that are associated with the Kalahari ecosystem (Spottiswoode 2005). We studied sociable weavers from 23 colonies at Benfontein Game Farm, Kimberley, South Africa (28°52′ S, 24°50′ E), in September–December 2010 and 2011, and January–February 2013, which was largely outside their breeding season. The majority of birds in this population have been captured annually since 1993 (except 2007). We used these captures to estimate the age of individuals from the first time they were captured. Sociable weavers may live up to 16 years, with 15.3% of all dispersing birds dispersing in their first calendar year and 69.2% before the age of three (REvD & BJH unpublished data). Sociable weavers live in colonies varying in size from five to over 300 individuals that occupy a communal nest that is built and maintained by the colony members. We assigned colour-ringed individuals to labelled nest chambers and scored thatch-building activity and location. Nest chambers are used throughout the year for roosting and breeding by family groups or, more rarely, by single individuals. Older birds are more likely to breed than younger ones, and the most frequently used nest chamber during the non-breeding season is generally used for breeding. Individuals entering multiple nest chambers may roost, build and seek refuge from predators in any of them (Maclean 1973; Collias & Collias 1977; van Dijk *et al.* 2013). The duration and number of our observations per colony varied depending on levels of activity and size of the colony. We stopped observing a given colony once additional observations provided no new information on the identity of thatch-builders and nest chamber assignment. To test whether individuals build the thatch near their own chamber, we used digital photographs and drawings from the underside of 14 communal nests to assess the 25% of nest chambers that were nearest to the one occupied by the thatch-builder. We then scored for each thatch-building event whether or not it had taken place above those nest chambers nearest to the thatch-builder's own nest chamber. If a thatch-builder built at multiple locations or if the thatch-builder used multiple nest chambers, we calculated the mean percentage of thatch-building at a given location weighted for the frequency of thatch-building at that location and weighted for the number of times the nest chamber was visited. Distances between nest chambers within each communal nest were measured using digital photographs of the underside of communal nests in Adobe Photoshop v. 7.0 (Adobe Systems Inc., San Jose, CA, USA).

### Genetic analysis

Blood samples (*ca*. 50 μL) were collected from the brachial vein using a sterile needle and heparinized capillary tube and were preserved in 1 mL of absolute ethanol. Genomic DNA was extracted using an ammonium acetate precipitation method (Richardson *et al.* 2001) in preparation for polymerase chain reaction amplification. The DNA content of the extractions was quantified using a Nanodrop ND8000 and diluted to a final approximate concentration of 15 ng μL^−1^. Sex was determined using the *P2*–*P8* sex-typing primers (Griffiths *et al.* 1998). Each sample was genotyped using 17 autosomal polymorphic microsatellite markers (mean ± SD = 9.5 ± 4.8 alleles; Table S1). Heterozygotes were observed for males and females at all genotyped loci indicating they were autosomal in sociable weavers. All genotyping scores were validated manually by including four standard samples on each plate and adjusted wherever the genotype call made by the software was deemed to be in error based on the shape, peak height (relative fluorescent units) and expected fragment size of the cloned sequence allele. We found an allelic error rate of 0.7% based on 110 (9.7%) re-extracted and re-genotyped randomly selected individuals (Pompanon *et al.* 2005). We used CERVUS v. 3.0.35 (Kalinowski *et al.* 2007) to quantify the number of alleles, calculate observed and expected heterozygosities and to estimate the frequency of null alleles. In total, 161 alleles were detected across 17 microsatellite markers and 1138 individuals genotyped. None of the 17 markers showed significant deviation from Hardy–Weinberg equilibrium (Table S1) or showed significant linkage disequilibrium after false discovery rate correction. Queller and Goodnight's genetic estimate of pairwise relatedness *r*_QG_, implemented in KINGROUP v. 2_090501 (Konovalov *et al.* 2004), was calculated with reference to genotypes from the entire population across all colonies (*n *=* *1138 birds). This is justified because both juvenile (see above) and adult dispersal is regular (11.9% of adults ringed in our population since 1993 dispersed at least once, while each adult may disperse 1.17 ± 0.42 times; REvD & BJH unpublished data), so that specific alleles may occur anywhere in our population. Furthermore, relatedness estimates based on reference to individuals within each colony are inappropriate given the size of study colonies (number of birds caught at colonies: mean ± SD = 31.7 ± 27.7, range = 7–128). Although the genetic composition of the population may vary over time due to various demographic processes such as dispersal, mortality and recruitment, such temporal segregation of individuals is unlikely to be problematic for our estimates of pairwise relatedness, which are based on data collected within 3 years. Sociable weavers may live up to 16 years, so the vast majority of individuals included in our study will have overlapped in time. In a conservative analysis of a single year (2010), relatedness estimates at the population and colony level using an alternative genotype-analysis software (SPAGeDi v. 1.4; Hardy & Vekemans 2002) were similar to those reported here (Appendix S1), showing that our estimates of relatedness are robust against potential temporal sampling effects. Further details of genetic analysis are described in the Appendix S1.

### Social network analysis

An individual was assigned to a given nest chamber once it had been observed to enter it, irrespective of the activity carried out, i.e. either building the nest chamber or roosting in it. A network ‘edge’ was drawn between individuals that used the same nest chambers either for roosting or nest-building at any given time within a series of observations at the same colony in the same year, either together in the nest chamber at the same time or at different times. These individuals were thus assumed to be associated. We note that our results do not depend on *a priori* assumptions about the social groupings of individuals based on the network, which can be problematic when using a ‘gambit of the group’ approach in exploratory social network analyses (Croft *et al.* 2008). The gambit of the group assumes that individuals are associated based on social group membership, *i.e*. it draws an edge between individuals that occur in the same social group. As such it provides a potentially useful description of their social organization from which predictions may be derived. However, as a network structure is highly sensitive to missing data and, for example, the weighting of edges, this approach may be misleading. In contrast to many previous studies (Croft *et al.* 2011; Pinter-Wollman *et al.* 2014), we instead use the network structure as an analytical means to test our independent, *a priori* hypotheses. The Girvan–Newman algorithm is an iterative process that uses the edge betweenness centrality of all edges to find partitions of the original data and identify social groups (‘Girvan–Newman-partitions’; Girvan & Newman 2002). We used the number of Girvan–Newman-partitions with the highest value of Newman and Girvan's modularity Q (Newman & Girvan 2004). We used Quadratic Assignment Procedure (QAP) with 5000 permutations to calculate the Pearson's correlation coefficient *R* and its probability *P* for the correlation between matrices of genetic relatedness and matrices of weighted network associations.

Social network analyses were performed for each colony separately. Short-term inter-colony visits are uncommon and usually brief, but any new immigrants from apparently permanent between-colony dispersal events were included in the colony-based social networks (REvD & BJH, unpublished data; Maclean 1973). Network centrality metrics Freeman degree *k* and Freeman betweenness *B* were based on undirected networks, weighted for association strength, and normalized to allow comparison between networks of different sized colonies (Croft *et al.* 2008). Network analyses were performed using Ucinet v. 6.37718 for Windows and Netdraw Network Visualization v. 2.09719 for Windows (Analytic Technologies, 2002; Harvard, MA, USA).

### Statistical analysis

To test whether node-based network metrics degree *k* and betweenness *B* differed between males and females, or between thatch-builders and non-builders, and to test whether network centrality was associated with genetic relatedness, we used resampling and Monte Carlo simulations with 10 000 iterations to account for the non-independence of data of node-based network metrics (Croft *et al.* 2008). We used Linear Mixed Models (LMM) in the package nlme for R (Pinheiro *et al.* 2010) to account for the statistical non-independence of data originating from a given colony and of individuals within colonies. Genetic relatedness and age estimates were log- and log+1-transformed, respectively, to achieve normality of the residuals of the linear mixed models. We entered colony and individual identity (where appropriate) as random factors with individual identity nested within colony. All of our initial models included colony size (i.e. the number of birds caught at the colony), year and observer (where appropriate). We used the Akaike Information Criterion for model selection. For the LMM testing whether Girvan–Newman partitioning predicted genetic relatedness between individuals, we removed two outliers from one individual with extremely low relatedness values (namely −0.641 and −0.627) from our final models to improve the model's fit to the data. The inclusion or removal of these outliers in our models did not qualitatively change the results. Our final model included the fixed factors whether individuals occurred within the same partition and whether individuals were of the same sex or not. The latter contributed significantly to the model (0.015 ± 0.005, *t *=* *3.050, d.f. = 3733, *P *=* *0.002, *n *=* *314 pairs). We therefore constructed separate models for each sex. To improve the model's fit to the data, the same two outliers as above were also removed from the model testing whether kin were spatially structured within communal nests. Again, this did not qualitatively change our results. Statistical analysis was performed using R version 2.12.1 (R Development Core Team 2010), except for resampling techniques and Monte Carlo simulations, which were applied using PopTools v. 3.2.5 in Excel(Hood 2010).

## Results

Dyadic relatedness differed between levels of social organization and between sexes. Relatedness among all birds, females and especially males, was significantly higher within colonies than at the population level (Table 1a). This male-biased genetic structure is a consequence of male philopatry, but even among males mean colony-level relatedness was low (*r *=* *0.055). The age of birds was not associated with relatedness to the rest of the colony (all: model effect estimate ± SE = 0.008 ± 0.006, *t *=* *1.458, d.f.* *= 342, *P *=* *0.146, *n *=* *366 birds in 23 colonies; males: 0.010 ± 0.007, *t *=* *1.361, d.f. = 195, *P *=* *0.175, *n *=* *219; females: 0.005 ± 0.009, *t *=* *0.566, d.f.* *= 132, *P *=* *0.573). Estimated relatedness between males within colonies decreased significantly with increasing distance between their nest chambers (−0.005 ± 0.001, *t *=* *5.618, d.f.* *= 378, *P *<* *0.001, *n *=* *505 males in 23 colonies). By contrast, there was no significant effect of inter-nest chamber distance on female relatedness (0.000 ± 0.001, *t *=* *0.158, d.f.* *= 157, *P *=* *0.874, *n *=* *236 females in 20 colonies). Thus, both males and females exhibited higher levels of relatedness within colonies than between colonies, and within colonies male relatives were spatially clustered.

**Table 1:** Mean ± SD relatedness estimates. Relatedness between all individuals, males and females (a) at the level of population and colony, and (b) at the level of social unit within colony [within Girvan–Newman partitions (‘within’) or between Girvan–Newman partitions (‘between’) in the same colony].

(a)	Population	Colony
All (1138)	−0.001 ± 0.151	0.032 ± 0.175***
Male (561)	0.000 ± 0.150	0.055 ± 0.191***
Female (530)	−0.002 ± 0.149	0.019 ± 0.162**

	Girvan–Newman partitions	
(b)	Within	Between
All (314)	0.054 ± 0.190	0.025 ± 0.169***
Male (174)	0.120 ± 0.213	0.036 ± 0.182***
Female (119)	0.035 ± 0.161	0.015 ± 0.156^n.s.^

Comparisons between mean relatedness of the entire population and those at the level of colony were made using one-sample Wilcoxon singed rank tests with the mean relatedness at the population level set as μ. Numbers in parentheses indicate samples size of the genotyped population. See text for statistics of Girvan–Newman partitions.

** *P *<* *0.01

*** *P *<* *0.001; n.s., not significant.

**Figure 2:**
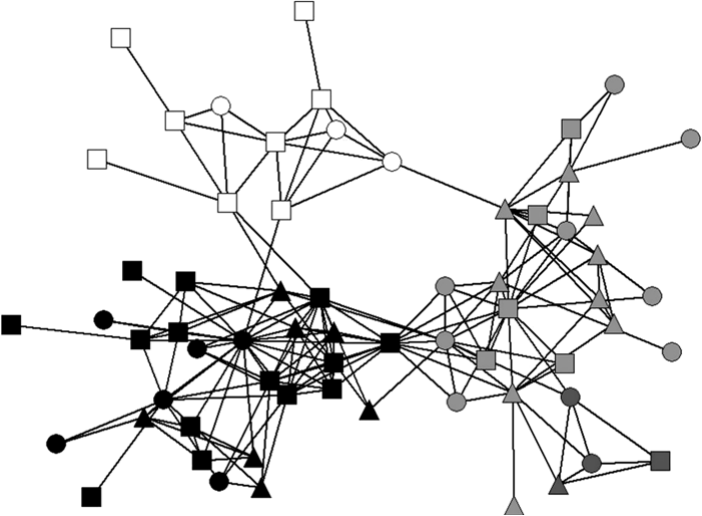
Example of a colony social network. Each node represents an individual, each edge connects individuals that used the same nest chamber. Squares represent males, circles females and triangles individuals of unknown sex. The different colours indicate the four Girvan-Newman partitions of this colony (four isolated individuals without any connections are excluded here).

To determine whether this spatial kin structure was reflected in social association, and hence has potential functional consequences, we used social network analysis. This approach quantifies complex interactions among individuals at the level of groups (Krause *et al.* 2007; Croft *et al.* 2008; Rand *et al.* 2011), and thus permits investigation of the role of kinship in driving social behaviour (Wolf & Trillmich 2008; Croft *et al.* 2012). We constructed social networks from observations of shared nest chamber use by 481 individuals at 23 colonies entering nest chambers 3175 times (mean = 6.6 ± 9.5 SD observations per bird). Multiple nest chambers were used by 308 (64%) birds, each entering 2.8 ± 2.4 (range: 1–19) nest chambers. Girvan–Newman partitioning showed that colony networks were divided into 4.3 ± 2.0 social groups or ‘Girvan–Newman-partitions’ (range: 2–9; modularity *Q *=* *0.263 ± 0.225, −0.200–0.647; Fig. 2). Thus, functional social groups were identifiable within colonies.

Girvan–Newman-partitions were predicted by kinship. Males associated within Girvan–Newman-partitions were significantly more related than those from the same colony that were not associated socially (0.073 ± 0.012, *t *=* *6.201, d.f.* *= 1179, *P *<* *0.001, *n *=* *174 male pairs; Table 1b). In contrast, female relatedness did not differ significantly within and between Girvan–Newman partitions (*P *=* *0.149, *n *=* *119 female pairs; Table 1b). When males and females were combined in one model to test whether Girvan–Newman-partitions were predicted by kinship, we found that relatedness within sexes was positively associated with classification within the same Girvan–Newman partition (0.053 ± 0.009, *t *=* *5.912, d.f. = 1745, *P *<* *0.001, *n *=* *293), whereas between sexes no such association was found (−0.003 ± 0.008, *t *=* *0.311, d.f. = 1706, *P *=* *0.756, *n *=* *303). These results did not change qualitatively when we restricted analyses to colonies with a strongly clustered structure (i.e. *Q *>* *0.3 for 10 colonies; Newman & Girvan 2004). Likewise, using the QAP for matrix correlations between pairwise relatedness and network associations, the mean correlation coefficient was significantly > 0 for males (Pearson's *R *=* *0.294; *t *=* *4.585, d.f.* *= 20, *P *<* *0.001), but not females (*R *=* *−0.005; *t *=* *0.081, d.f.* *= 14, *P *=* *0.936; Table S2). Controlling for spatial clustering of individuals using Multiple Regression QAP (Dekker *et al.* 2007) had no qualitative effect on these results: the regression coefficient was significantly > 0 for males (β* *= 0.944 ± 0.424; *V *=* *185, *P *=* *0.014, *n *=* *21), but not females (β* *= −0.123 ± 0.196; *V *=* *22, *P *=* *0.625, *n *=* *10), showing that male kin associate irrespective of their spatial clustering. Therefore, the communal nests are spatially and socially structured with respect to male, but not female relatedness. We now consider whether cooperative investment reflected this social structure.

Males dominated thatch-building: 71.8% of 142 marked birds observed building were male and they contributed a disproportionate 88.6% of 1323 thatch-building events (χ^2^ = 20.1; d.f.* *= 1, *P *<* *0.001). An individual's position within its social network predicted communal investment. The network centrality metrics degree *k* (index of an individual's gregariousness) and betweenness *B* (index of the inter-node paths passing through an individual node) were significantly higher for thatch-building birds than expected by chance (both *P ≤ *0.001, *n *=* *185 thatch-builders and 269 non-builders). When we analysed network centrality separately for each sex, k and *B* were significantly higher than expected by chance for male builders (*P *=* *0.007, *P *=* *0.027, respectively, *n *=* *204–102 thatch-building and 102 non-building males), while for females there was a similar pattern (*P *=* *0.077, *P *=* *0.050, respectively, *n *=* *140–40 thatch-building and 100 non-building females). Furthermore, relatedness of an individual to other colony members was correlated with both network centrality measures (Spearman correlations: *k*, *R*_s_ = 0.102, *P *=* *0.029, *n *=* *348; *B*, *R*_s_ = 0.189, *P *=* *0.013, *n *=* *348). Additionally, we found that the relatedness of individual males and females to other colony members was also correlated with both network centrality measures, with *k* showing a near significant, positive trend in both sexes (males: *R*_*s*_ = 0.111, *P *=* *0.059, *n *=* *205; females: *R*_*s*_ = 0.026, *P *=* *0.060, *n *=* *133), while *B* was significantly, positively associated with relatedness (males: *R*_*s*_ = 0.195, *P *=* *0.025, *n *=* *205; females: *R*_*s*_ = 0.132, *P *=* *0.027, *n *=* *133). Age did not predict network centrality (*k*: *R*_*s*_ = −0.015, *P *=* *0.771, *n *=* *398; *B*: *R*_*s*_ = 0.167, *P *=* *0.065, *n *=* *398). Thus, social substructure within colonies was related to communal investment and kinship in both sexes.

**Figure 3:**
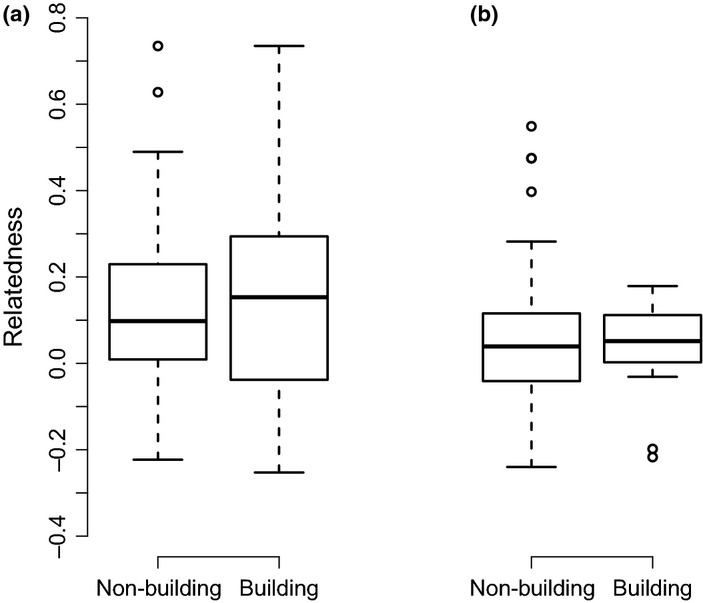
Relatedness among 25% nearest neighbours of thatch-building vs. non-building sociable weavers. (a) within males (*n *=* *262), and (b) within females (*n *=* *138). Box plots indicate the median, the interquartile range, the maximum and minimum values excluding outliers, and outliers.

Finally, we examined whether the spatial structure of relatives within communal nests predicted thatch-building. We found that 60.8% of thatch-building occurred within the quartile of the communal nest closest to individual builder's own nest chambers, i.e. more frequently than expected by chance (*V *=* *11457.5, *P *<* *0.001, *n *=* *162 individuals, *μ *= 0.25). This indicates self- and kin-directed communal investment because building near one's own nest chamber also benefits spatially clustered relatives. Furthermore, although birds observed building were not more closely related to other colony members than non-builders (−0.016 ± 0.010, *t *=* *1.517, d.f. = 318, *P *=* *0.130), there was a significant interaction with sex (0.024 ± 0.011, *t *=* *2.201, d.f. = 318, *P *=* *0.028), showing that male (but not female) builders had above-average relatedness to the rest of the colony (Fig. S1). This result did not change qualitatively when age was included as a covariate in this model (age: 0.002 ± 0.002, *t *=* *1.259, d.f. = 317, *P *=* *0.209). Importantly, the effect of kinship on communal investment was a function of local rather than general relatedness within colonies. Although thatch-building was not associated with relatedness at the level of colony for either sex (males: 0.010 ± 0.012, *t *=* *0.877, d.f. = 177, *P *=* *0.382; female: 0.008 ± 0.014, *t *=* *0.583, d.f. = 123, *P *=* *0.561), relatedness of males occupying the 25% of a colony's nest chambers nearest to a focal male (i.e. where thatch-building is primarily directed) was significantly higher for nest-builders than for non-builders (0.037 ± 0.015, *t *=* *2.515, d.f. = 374, *P *=* *0.012; Fig. 3; Table S3a). No such effect was observed in females (0.001 ± 0.021, *t *=* *0.041, d.f. = 374, *P *=* *0.968; Table S3b). Age was not associated with local relatedness among males (*P *=* *0.540) or females (*P *=* *0.651) and the results of our models of local relatedness predicting thatch-building remained qualitatively unchanged when age was included (male age: 0.001 ± 0.002, *t *=* *0.465, d.f. = 373, *P *=* *0.642; female age: 0.001 ± 0.002, *t *=* *0.725, d.f.* *= 373, *P *=* *0.469). Therefore, communal investment was not influenced by colony-level relatedness, but was a function of relatedness among males at the local level, where thatch-building benefits self and kin.

## Discussion

Using observations of behaviour and fine-scale population genetic structure, we have shown that male kin are spatially and socially clustered within the massive communal nests of sociable weavers, and that the construction and maintenance of public goods in this species is predicted by the presence of kin despite the low average relatedness among members of a colony. Although high levels of relatedness, such as that within nuclear and extended families, are not required for kin selection to operate, previous support for kin selection as a mechanism to promote cooperation stems mainly from systems where relatedness is high within discrete social groups. Our study demonstrates that kin selection may also operate in groups with much lower average levels of relatedness as a result of the spatial and social organization of relatives within those groups, and conditional expression of cooperative behaviour according to the presence of kin.

The ecology of animals often plays a critical role in conflict resolution by determining the extent to which individuals interact with relatives or with the same social partners in repeated events. In bacteria, for example, the physical conditions of natural habitats, such as the interaction between the existence of biofilms and fluid flow, have been shown to influence the risk of exploitation of a public good (Drescher *et al.* 2014), and in humans, various demographic and ecological components of the social environment influence the prevalence of cooperation (Lamba & Mace 2011; Apicella *et al.* 2012). The ecology of sociable weavers is also likely to be an important driver behind their communal lifestyle. Their large, thatched communal nest provides both a thermoregulatory buffer against the harsh and variable ambient temperatures (van Dijk *et al.* 2013) and a refuge from predators. These functions require communal construction and maintenance to retain the integrity of the nest mass and provide support for individual nest chambers, and the spatial structuring of relatives means that construction of thatch above one's own nest chamber will also provide these benefits for relatives' nest chambers. This opportunity for social interactions among kin arises from the limited dispersal of males, resulting in strong kin-structure between and within colonies. Thus, the incentive to cooperate is not simply a function of individuals' social environment; instead, the evolution of cooperative investment in public goods and the resolution of a potential tragedy of the commons can be viewed as a consequence of the ecological factors that select for natal philopatry.

Our finding that thatch-building occurs near an individual's own nest chamber might be a result of social dynamics within colonies. Individuals that attempt to build the thatch elsewhere may, for example, experience aggression from more distant colony members. Whether such social interactions could drive self- and kin-directed thatch-building is something we cannot test directly, because thatch-building away from a focal bird's own nest chamber is infrequent and aggressive interactions during thatch-building rare. We note, however, that if social interactions at colonies do determine thatch-building location, this may provide the social mechanism that results in kin-biased behaviour, and our proposition that thatch-building is kin-directed and its benefits are shared with kin would still be supported.

One alternative explanation is that the shared benefits of thatch-building are a by-product of selfish investment in the communal thatch. Because relatives happen to live near a builder's own nest chamber, they may share the benefits of the builder's investment simply as a selfish by-product. However, this explanation is unlikely for two reasons. First, our result that thatch-building effort was positively associated with local relatedness is inconsistent with the suggestion of a selfish by-product. Second, we have shown that network associations are predicted by relatedness, irrespective of the distance between nest chambers, suggesting that active discrimination of kin promotes the benefits of sharing a communal nest with relatives, rather than this being a passive process.

Two further alternative explanations for investment in the communal thatch of sociable weavers can be disregarded. First, work might be enforced, defectors being punished by other colony members. This is unlikely because most thatch-building is done by a relatively small number of adults at each colony (38.5 ± 23.1% of adults were observed to build at least once at each colony), so a large proportion of adults benefited from the communal thatch without directly contributing to it. Although not all defectors need to be punished in order for enforcement to operate, the problem in the large colonies of sociable weavers is that the investment in thatch-building and punishment of defecting individuals would be hard to police. This explanation also suffers from the second-order problem of cheating, i.e. who bears the costs of enforcement (Hauert *et al.* 2007)? Second, the tragedy of the commons is easily resolved if there are selfish, direct benefits of cooperation. For example, cooperative behaviour could be a sexually selected signal (Zahavi 1995), explaining why it is most commonly observed in males. Again, this explanation is unlikely because most pairs remain together for multiple years (R. Covas & C. Doutrelant, pers. comm.) and extra-pair paternity is rare or absent in sociable weavers (Covas *et al.* 2006), suggesting that the intensity of sexual selection is relatively low. Moreover, only 50% of all males were observed thatch-building, and thatch-building birds were significantly older than expected by chance (*P *=* *0.005, *n *=* *377), while the need to attract a mate is probably highest for young birds that have not yet paired. Furthermore, it is difficult to reconcile this explanation with the fact that thatch-building is associated with the local relatedness of builders.

In conclusion, our results are consistent with the idea that population viscosity promotes the expression of kin-directed cooperation (Lion & van Baalen 2008; Hatchwell 2010). Kin-directed cooperative behaviour has usually evolved among relatives living in discrete family groups, but here, we have shown that despite low colony-level relatedness, fine-scale kin neighbourhoods exist among the communal nests of sociable weavers, a pattern that is reflected in their social interactions. Most importantly, we have shown for the first time that investment in a communal structure that provides shared benefits to colony members, and can hence be regarded as a public good, is kin-directed and thus potentially a product of kin selection despite the large size and low average relatedness of colonies.
