# Supplementary material for: Cooperative investment in public goods is kin directed in communal nests of social birds
Source: Ecol Lett. 2014 Jul 6;17(9):1141–8. doi: 10.1111/ele.12320 (PMC4282064; doi:10.1111/ele.12320)
Supplement: Supplementary file 1 [file ele0017-1141-SD1.docx]

**Figure S1** Colony-level relatedness of thatch-building versus non-building sociable weavers. a, males (n = 198), and b, females (n = 142). Box plots indicate the median, the interquartile range, the maximum and minimum values excluding outliers, and outliers.

(a) (b)
