# Supplementary material for: Cooperative investment in public goods is kin directed in communal nests of social birds
Source: Ecol Lett. 2014 Jul 6;17(9):1141–8. doi: 10.1111/ele.12320 (PMC4282064; doi:10.1111/ele.12320)
Supplement: Supplementary file 2 [file ele0017-1141-SD2.docx]

**Table S1** Summary statistics of 17 microsatellite markers polymorphic in the sociable weaver

|  |  |  |  |  |  |  |  |  | | Primer sequence (5’ – 3’) | | |  |
| --- | --- | --- | --- | --- | --- | --- | --- | --- | --- | --- | --- | --- | --- |
| Locus | *N* | *N*_a_ | *H*_O_ | *H*_E_ | *P*_HWE_ | Allele size range (bp) | Fluorescent dye | Multiplex set | Forward | | | Reverse | Reference |
| *GCSW15* (*43*) | 44 | 7 | 0.77 | 0.77 | 0.701 | 162-183 | HEX | 1 | GGACGACTCCTTTATTTCCC | | TTCTGACTTCCYCAGGTAACAC | | McRae *et al.* 2005 |
| *GCSW47* (*43*) | 43 | 5 | 0.54 | 0.60 | 0.901 | 199-210 | 6-FAM | 1 | GGCTTCTCTGGTTGCATGTC | | ACAGTAATCCCCAGCCATCA | | McRae *et al.* 2005 |
| *INDIGO40* (*44*) | 42 | 5 | 0.67 | 0.77 | 0.609 | 207-215 | NED | 1 | ACCGAAACAACAGAAACAGT | | AGAACGCTAAGTGAATGTCC | | Sefc *et al.* 2001 |
| *TG22-001* (*45*) | 42 | 6 | 0.67 | 0.70 | 0.434 | 248-266 | HEX | 1 | TTGGATTTCAGAACATGTAGC | | TCTGATGCAAGCAAACAA | | Dawson *et al.* 2010 |
| *GCSW35* (*43*) | 45 | 10 | 0.76 | 0.81 | 0.107 | 162-246 | HEX | 2 | AAATGATTGCCACTCCATGA | | AACCTGTTCCTGGTCACACC | | McRae *et al.* 2005 |
| *INDIGO41* (*44*) | 44 | 7 | 0.89 | 0.78 | 0.138 | 204-324 | 6-FAM | 2 | GACAGTGTTCAGGAGAAGATAC | | TTTCCTACAGGATTCCCTAC | | Sefc *et al.* 2001 |
| *Ppi2-Gga* | 44 | 6 | 0.73 | 0.70 | 0.225 | 180-211 | 6-FAM | 2 | GGACCCTGTAACAGAAACCA | | CCAGCAGAGGGCATGT | | this study |
| *TG01-148* (*45*) | 44 | 3 | 0.64 | 0.62 | 1.000 | 195-201 | NED | 2 | TTGCAACACATTCTAATATTGC | | TTTAAAGTACATCAAACAACAAAATC | | Dawson *et al.* 2010 |
| *WBSW9* (*46*) | 45 | 13 | 0.78 | 0.86 | 0.049 | 108-246 | 6-FAM | 2 | TTGAGTGGCTAATTTTGTGAAGG | | TCTTATGTCCCCCATTTGGA | | McRae & Amos 1999 |
| *CAM-01* (*47*) | 45 | 14 | 0.73 | 0.85 | 0.398 | 197-328 | HEX | 3 | AAAGGCCAAGRCCAGTATG | | CTCTCATCCACCCTGTTAGC | | Dawson *et al.* 2013 |
| *CAM-15* (*47*) | 45 | 10 | 0.73 | 0.82 | 0.213 | 110-283 | NED | 3 | SGACGACTCCTTTATTTCCC | | TTCTGACTTCCYCAGGTAACAC | | Dawson *et al.* 2013 |
| *GCSW13* (*43*) | 45 | 8 | 0.62 | 0.71 | 0.071 | 154-308 | HEX | 3 | AAGGCTGTAGAAACACCTTACA | | TGTCTAACTTTAGTCAACATAATGC | | McRae *et al.* 2005 |
| *INDIGO29* (*44*) | 45 | 16 | 0.82 | 0.86 | 0.283 | 184-238 | 6-FAM | 3 | TCAGGGAGCAAATCTCTACG | | GGAAGAAGGCTGGGTAAAAT | | Sefc *et al.* 2001 |
| *Ase18* (*48*) | 45 | 9 | 0.73 | 0.71 | 0.321 | 183-236 | NED | 4 | ATCCAGTCTTCGCAAAAGCC | | TGCCCCAGAGGGAAGAAG | | Richardson *et al.* 2000 |
| *GCSW31* (*43*) | 45 | 15 | 0.87 | 0.86 | 0.479 | 300-344 | 6-FAM | 4 | GCACAGACACACTGCTACTGG | | CTTTGTAGCATGAGGACAGCA | | McRae *et al.* 2005 |
| *GCSW57* (*43*) | 45 | 21 | 0.76 | 0.93 | 0.061 | 149-266 | HEX | 4 | TTGCTGTTCTTCCACACTGC | | TCAGATGGGTGAATTTCTTGG | | McRae *et al.* 2005 |
| *TG07-022* (*45*) | 40 | 6 | 0.50 | 0.55 | 0.459 | 418-435 | HEX | 4 | CAGAAGACTGTGTTCCTTTTGTTC | | TTCTAATGTAGTCAGCTTTGGACAC | | Dawson *et al.* 2010 |

The markers genotyped included several isolated in species closely related to sociable weaver (*GCSW13*, *GCSW15*, *GCSW31*, *GCSW35*, *GCSW47*, *GCSW57*, *INDIGO29*, *INDIGO40*, *INDIGO41* and *WBSW9*), and others of known high cross-species utility (*Ase18*, *CAM-01*, *CAM-15*, *Ppi2-Gga*, *TG01-148*, *TG03-098*, *TG07-022* and *TG22-001*). Statistics are based on a genotyping group of 45 unrelated individuals sampled from our population. *N*, number of unrelated individuals genotyped; *N_a_*, number of alleles observed; *H*_O_, observed heterozygosity; *H*_E_, expected heterozygosity; *P*_HWE_, probability of deviation from Hardy-Weinberg equilibrium (none were significant after FDR correction). Primer designed following previously published methods (Kuepper *et al.* 2008).

REFERENCES

Dawson, D.A., Ball, A.D., Spurgin, L.G., Martín-Gálvez, D., Stewart, I.R.K., Horsburgh, G.J.*, et al.* (2013). High-utility conserved avian microsatellite markers enable parentage and population studies across a wide range of species. *BMC Genomics*, 14, 176.

Dawson, D.A., Horsburgh, G.J., Kuepper, C., Stewart, I.R.K., Ball, A.D., Durrant, K.L.*, et al.* (2010). New methods to identify conserved microsatellite loci and develop primer sets of high cross-species utility – as demonstrated for birds. *Mol. Ecol. Resour.*, 10, 475-494.

Kuepper, C., Burke, T., Szekely, T. & Dawson, D.A. (2008). Enhanced cross-species utility of conserved microsatellite markers in shorebirds. *BMC Genomics*, 9, 502.

McRae, S.B. & Amos, W. (1999). Characterization of hypervariable microsatellites in the cooperatively breeding white-browed sparrow weaver *Plocepasser mahali*. *Mol. Ecol.*, 8, 903-904.

McRae, S.B., Emlen, S.T., Rubenstein, D.R. & Bogdanowicz, S.M. (2005). Polymorphic microsatellite loci in a plural breeder, the grey-capped social weaver (Pseudonigrita arnaudi), isolated with an improved enrichment protocol using fragment size-selection. *Mol. Ecol. Notes*, 5, 16-20.

Richardson, D.S., Jury, F.L., Dawson, D.A., Salgueiro, P., Komdeur, J. & Burke, T. (2000). Fifty Seychelles warbler (*Acrocephalus sechellensis*) microsatellite loci polymorphic in Sylviidae species and their cross-species amplification in other passerine birds. *Mol. Ecol.*, 9, 2225-2230.

Sefc, K.M., Payne, R.B. & Sorenson, M.D. (2001). Characterization of microsatellite loci in village indigobirds Vidua chalybeata and cross-species amplification in estrildid and ploceid finches. *Mol. Ecol. Notes*, 1, 252-254.
