# Supplementary material for: Cooperative investment in public goods is kin directed in communal nests of social birds
Source: Ecol Lett. 2014 Jul 6;17(9):1141–8. doi: 10.1111/ele.12320 (PMC4282064; doi:10.1111/ele.12320)
Supplement: Supplementary file 3 [file ele0017-1141-SD3.docx]

**Table S3** Nest-building in response to relatedness among spatially clustered males and females, (a) accounting for relatedness within males or between males and females, and (b) for relatedness within females or between males and females.

(a)

|  | Model effect estimate ± SE | *df* | *t* | *P* |
| --- | --- | --- | --- | --- |
| Distance* | -0.001 ± 0.000 | 374 | 1.813 | 0.071 |
| Builder† | -0.023 ± 0.009 | 374 | 2.651 | 0.008 |
| Within-male relatedness‡ | 0.027 ± 0.009 | 374 | 2.928 | 0.004 |
| Builder*Within-male relatedness | 0.037 ± 0.015 | 374 | 2.515 | 0.012 |

*****Distance between nest chambers. †Factor indicating whether an individual was observed nest building or not. ‡Factor indicating relatedness within males or between males and females.

(b)

|  | Model effect estimate ± SE | *df* | *t* | *P* |
| --- | --- | --- | --- | --- |
| Distance* | -0.001 ± 0.000 | 374 | 2.112 | 0.035 |
| Builder† | -0.005 ± 0.008 | 374 | 0.581 | 0.561 |
| Within-female relatedness‡ | -0.006 ± 0.010 | 374 | 0.581 | 0.562 |
| Builder*Within-female relatedness | 0.001 ± 0.021 | 374 | 0.041 | 0.968 |

*Distance between nest chambers. †Factor indicating whether an individual was observed nest building or not. ‡Factor indicating relatedness within females or between males and females.
