# Supplementary material for: Cooperative investment in public goods is kin directed in communal nests of social birds
Source: Ecol Lett. 2014 Jul 6;17(9):1141–8. doi: 10.1111/ele.12320 (PMC4282064; doi:10.1111/ele.12320)
Supplement: Supplementary file 5 [file ele0017-1141-SD5.docx]

**Genetic analysis – additional details**

Sex was determined using the P2–P8 sex-typing primers (Griffiths *et al.* 1998) which were amplified as a singleplex reaction with the following PCR profile: 95˚C for 15 min, 30 cycles of 94˚C for 30 s, 48˚C for 45 s and 72˚C for 45 s, followed by a final extension step of 72˚C for 5 min. Each sample was genotyped using a QIAGEN Multiplex PCR Master Mix kit on 18 autosomal loci, which were grouped into four primer sets using MULTIPLEX MANAGER v.1.04 (Holleley & Geerts 2009; Table S1). Locus *TG03-098* (amplified in multiplex set 3) was excluded from further analyses as it displayed several alleles differing by only one base pair that could not be scored confidently. We used the following PCR profile for these four multiplexes: 95˚C for 15 min, 10 cycles of 94˚C for 30 s, 62˚C for 60 s (decreasing by 1˚C per cycle) and 72˚C for 90 s, followed by 25 cycles of 94˚C for 30 s, 52˚C for 60 s and 72˚C for 90 s and a final extension step of 72˚C for 10 min. PCR products were separated using an ABI3730 48-well capillary sequencer and the GeneScan™ 500 ROX™ Size Standard with prism set D (Applied Biosystems). Results were analysed using GENEMAPPER v3.7 (Applied Biosystems). We used GENEPOP v. 4.26 (Raymond & Rousset 1995) to check for Linkage Disequilibrium (LD) between loci and test each locus for conformity to Hardy–Weinberg equilibrium (HWE). For each test, the dememorisation number was set to 1000, the number of batches to 100 and the number of iterations per batch to 1000. *P*-values for LD and HWE were corrected using the false discovery rate (FDR) method (Verhoeven *et al.* 2005; Table S1).

To test for the robustness of our relatedness estimates against temporal sampling effects, we analysed genotypes from birds observed in 2010 only using an alternative software to KINGROUP, SPAGeDi (Hardy & Vekemans 2002). The results of this analysis are comparable to those reported in Table 1a in the main paper (Table SA1).

**Table SA1** Mean ± SE relatedness estimates. Relatedness between all individuals, males and females at the level of population and colony for birds observed in 2010 only.

|  |  |  |  |
| --- | --- | --- | --- |
|  | population | colony |  |
| all (628) | -0.002 ± 0.001 | 0.040 ± 0.004*** |  |
| male (337) | -0.001 ± 0.002 | 0.062 ± 0.007*** |  |
| female (280) | -0.004 ± 0.003 | 0.017 ± 0.005** |  |

A jackknife procedure over loci was used to estimate standard errors of genetic relatedness. Comparisons between mean relatedness of the entire population and those at the level of colony were made using one-sample t-tests with the mean relatedness at the population level set as μ. Numbers in parentheses indicate samples size of the genotyped population. ** *P* < 0.01, *** *P* < 0.001

REFERENCES

Griffiths, R., Double, M.C., Orr, K. & Dawson, R.J.G. (1998). A DNA test to sex most birds. *Mol. Ecol.*, 7, 1071-1075.

Hardy, O.J. & Vekemans, X. (2002). SPAGeDi: a versatile computer program to analyse spatial genetic structure at the individual or population levels. *Mol. Ecol. Notes*, 2, 618-620.

Holleley, C.E. & Geerts, P.G. (2009). Multiplex Manager 1.0: a crossplatform computer program that plans and optimizes multiplex PCR. *BioTechniques*, 46, 511-517.

Raymond, M. & Rousset, F. (1995). GENEPOP (version 1.2): population genetics software for exact tests and ecumenicism. *J. Hered.*, 86, 248-249.

Verhoeven, K.J.F., Simonsen, K.L. & McIntyre, L.M. (2005). Implementing false discovery rate control: increasing your power. *Oikos*, 108, 643-657.
